# Topology preserving stratification of tissue neoplasticity using Deep Neural Maps and microRNA signatures

**DOI:** 10.1186/s12859-022-04559-4

**Published:** 2022-01-13

**Authors:** Emily Kaczmarek, Jina Nanayakkara, Alireza Sedghi, Mehran Pesteie, Thomas Tuschl, Neil Renwick, Parvin Mousavi

**Affiliations:** 1 Medical Informatics Laboratory, School of Computing, Queen’s University, Kingston, Canada; 2 Laboratory of Translational RNA Biology, Department of Pathology and Molecular Medicine, Queen’s University, Kingston, Canada; 3 Department of Electrical and Computer Engineering, University of British Columbia, Vancouver, Canada; 4 Laboratory of RNA Molecular Biology, Rockefeller University, New York, USA

**Keywords:** Deep learning, miRNA, Cancer classification

## Abstract

**Background:**

Accurate cancer classification is essential for correct treatment selection and better prognostication. microRNAs (miRNAs) are small RNA molecules that negatively regulate gene expression, and their dyresgulation is a common disease mechanism in many cancers. Through a clearer understanding of miRNA dysregulation in cancer, improved mechanistic knowledge and better treatments can be sought.

**Results:**

We present a topology-preserving deep learning framework to study miRNA dysregulation in cancer. Our study comprises miRNA expression profiles from 3685 cancer and non-cancer tissue samples and hierarchical annotations on organ and neoplasticity status. Using unsupervised learning, a two-dimensional topological map is trained to cluster similar tissue samples. Labelled samples are used after training to identify clustering accuracy in terms of tissue-of-origin and neoplasticity status. In addition, an approach using activation gradients is developed to determine the attention of the networks to miRNAs that drive the clustering. Using this deep learning framework, we classify the neoplasticity status of held-out test samples with an accuracy of 91.07%, the tissue-of-origin with 86.36%, and combined neoplasticity status and tissue-of-origin with an accuracy of 84.28%. The topological maps display the ability of miRNAs to recognize tissue types and neoplasticity status. Importantly, when our approach identifies samples that do not cluster well with their respective classes, activation gradients provide further insight in cancer subtypes or grades.

**Conclusions:**

An unsupervised deep learning approach is developed for cancer classification and interpretation. This work provides an intuitive approach for understanding molecular properties of cancer and has significant potential for cancer classification and treatment selection.

## Background

Accurate cancer classification is the key to understanding and selecting effective treatments for this broad group of diseases. Typically, pathologists classify cancers using integrated morphologic, immunohistochemical, and/or targeted molecular analyses of tumor tissues. However, pathological diagnosis suffers from inter- and intra-observer variability [1]. Molecular analyses are becoming increasingly broad and/or “unbiased” due to the advent of -omics methodologies, including next-generation sequencing of cancer genomes and transcriptomes [2, 3].

microRNAs (miRNAs) are a class of small ($\sim$22 nt) regulatory RNA molecules and cancer classificatory markers that are readily detectable through next-generation sequencing and other approaches [4–7]. In humans and many other species, miRNAs negatively regulate gene expression through molecular interactions between the miRNA seed sequence (nucleotide positions 2–8) and complementary sites in target genes [8]. However, these molecules are also excellent biomarkers due to their tractable number ($\sim$1200 are encoded within the human genome), abundance, cell-type and disease-stage specificity, and stability in tissue and blood samples [5, 9, 10]. In addition, certain miRNAs have been shown to act as oncomiRs or tumour suppressors based on the function of their target gene [11–13]. These molecules are, therefore, highly valuable biomarkers for cancer research. Similar to other -omics data, miRNA datasets suffer from the curse of dimensionality (i.e. number of variables exceed that of observations), and there is a pressing need for building computational approaches that accurately derive clinically useful information from large data.

Computational methods that can effectively handle the challenges associated with large-scale data have evolved greatly in the past decade [14, 15]. In particular, machine learning has undergone major transformations including development of deep learning approaches that directly derive informative attributes and representations of large-scale data from its raw form [16]. Traditional machine learning methods require carefully engineered features of the raw data, and hence domain knowledge, as the specificity of the engineered features plays a crucial role in the success of algorithmic solutions. In contrast, deep learning directly uses the raw data to capture informative attributes representative of pathology or phenotypes of interest, making it well-suited to analyze combinatorial and complex relationships between miRNA expression and disease. However, there still exist certain limitations to miRNA-based deep learning for clinical implementation, including the current lack of interpretable methods [17]. In addition, to properly learn the features of raw data, deep learning performance depends on having a sufficiently high number of samples within the dataset.

To date, microRNA-based machine learning has been used to diagnose, classify, prognose, and stage various cancers with high accuracy [18–26]. Nanayakkara et al. [21] classify nine subtypes of neuroendocrine neoplasm using multiple layers of Support Vector Machines where each layer performs a specific classification task and they achieve an overall accuracy of 98%. Similarly, Ali et al. [18] achieve 95% accuracy in classifying kidney cancer subtypes using recurrent neural networks. The success of these studies demonstrates the potential role of miRNAs as cancer biomarkers. Most of these studies use supervised learning, requiring reliably labelled samples. Unsupervised learning is advantageous in finding similarities between samples based on characteristics (features) of each sample, as opposed to learning class labels. Exploration of cancer signatures using miRNA data and clustering techniques has been studied previously in the literature [27–31]. These studies have primarily focused on suggesting new cancer subtypes, or visualizing groups of already-known histological subtypes. Most of these papers, however, still use supervised learning approaches for any classification task. Few papers have used unsupervised deep learning for cancer classification with microRNA data. Liang et al. [32] use unsupervised learning through a Deep Belief Network with mRNA, miRNA, DNA methylation, and clinical attributes to cluster subtypes of ovarian and breast cancers, and identify important biomarkers with a two sample *t* test. Pyman et al. [33] use a mixed supervised and unsupervised deep model to classify neoplastic from non-neoplastic tissue. However, examination of fully unsupervised deep learning workflows for miRNA-based neoplastic tissue classification, along with quantitative approaches to study the underlying attention (i.e., identification of important/driving features of each sample) of the networks to miRNAs or samples is largely an under-explored area.

In this work, we present an end-to-end unsupervised method for representation learning and visualization of cancer and non-cancer samples using miRNA expressions from a large breadth of tissue types. In our approach, Deep Neural Maps (DNM) [34, 35] are used for representation learning in the form of autoencoders (AEs) to capture data manifolds in the latent features that accurately represent the original data. The latent features are then fed to a Self-Organizing Map (SOM) with a 2D lattice to group together similar tissue types in the form of topology preserving maps. The unsupervised learning workflow eliminates the need for reliably labelled samples, and bases clustering entirely on the molecular properties of the data. In addition, we perform joint optimization between the AE and SOM, generating a latent space specifically tailored for better topological mapping. We use an independent set of labelled data for testing the performance of our approach for classification of tissue-of-origin and neoplasticity status. Importantly, we implement a method based on activation gradients to evaluate the attention of our network to individual features. Our work is the first to examine tissue stratification and classification across a wide range of cancers through fully unsupervised deep learning. The activation gradients are particularly well-suited for this, which help identify previously unexplored molecular drivers of various cancers. Our work, therefore, has the potential to discover new molecular classifications and properties of cancers, leading to better understanding of their origin, and the selection of targeted treatments.

## Materials and methods

### Data acquisition and preprocessing

**Fig. 1:**
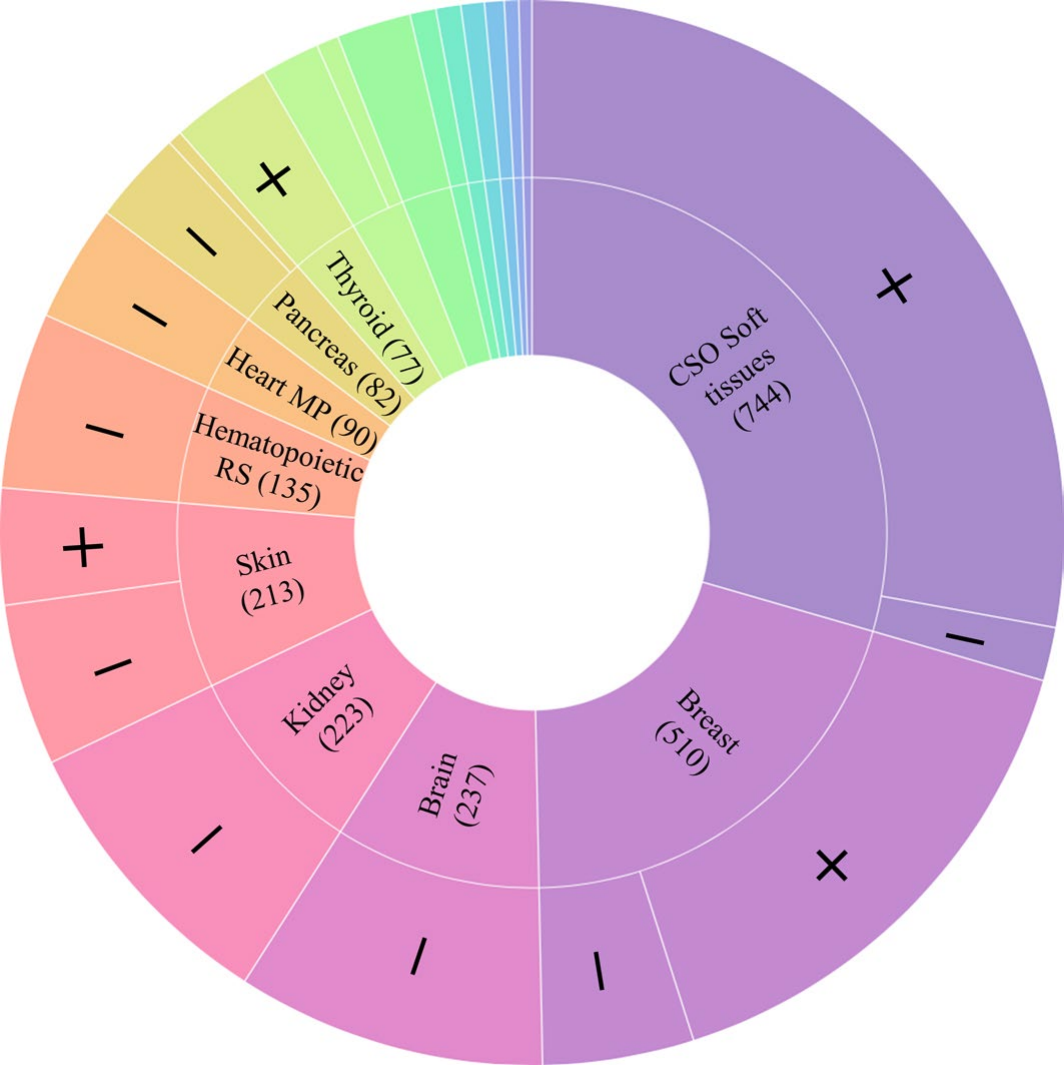
Sunburst diagram depicting sample ontology. The outermost layer represents the neoplasticity status, and the innermost the tissue-of-origin. In the labels, + represents neoplastic tissue, and - represents non-neoplastic tissue. Wedge size is proportional to the number of samples (shown in brackets). Eight class labels [Bronchus and lung (62), Endocrine RS (57), Adrenal gland (20), Intestine (20), Lymph node (18), Testis (15), Small intestine (11), and Paraganglion (10)] are omitted due to small wedge size. In total, there are 22 unique classes of combined tissue-of-origin and neoplasticity as shown in the outer ring. Only 5 tissues-of-origin contain both neoplastic and non-neoplastic samples (CSO Soft tissues, Breast, Skin, Pancreas, and Bronchus and Lung). *CSO Soft tissues* Connective, subcutaneous, and other soft tissues, *Endocrine RS* endocrine glands and related structures, *Heart MP* heart, mediastinum, and pleura, *Hematopoietic RS* hematopoietic and reticuloendothelial system

In this study, we access sample hierarchy data (tissue-of-origin, neoplasticity status) and comprehensive miRNA expression profiles from 2026 neoplastic and 1659 non-neoplastic tissue samples from an ongoing miRNA sequence curation and expression atlas project at Queen’s University, Canada and The Rockefeller University, US. Parts of the atlas data are available, including breast tissue samples [36] and neuroendocrine tumours and matched healthy tissue from a number of organs [21]. In addition to tissue-of-origin and neoplasticity status, the sample hierarchy includes disease subtypes (e.g., ductal carcinoma or triple-negative neoplastic breast tissue, melanoma or basal cell carcinoma of the skin, etc.), but are not considered in this study. miRNA expression profiles are quality controlled using an established data preprocessing approach [21], comprising miRNA expression normalization with total count scaling, removal of outlier samples with Interquartile Range (IQR) method [37], and filtering of samples with less than one million sequence reads. This results in a total of 984 miRNA features per sample. To facilitate computational method development, we also remove classes of combined tissue-of-origin and neoplasticity status with less than 10 samples. Following data preprocessing, miRNA expression profiles from 2524 samples are included in subsequent analyses below; sample ontology is depicted in Fig. 1 (created using [38]).

### Deep Neural Maps

**Fig. 2:**
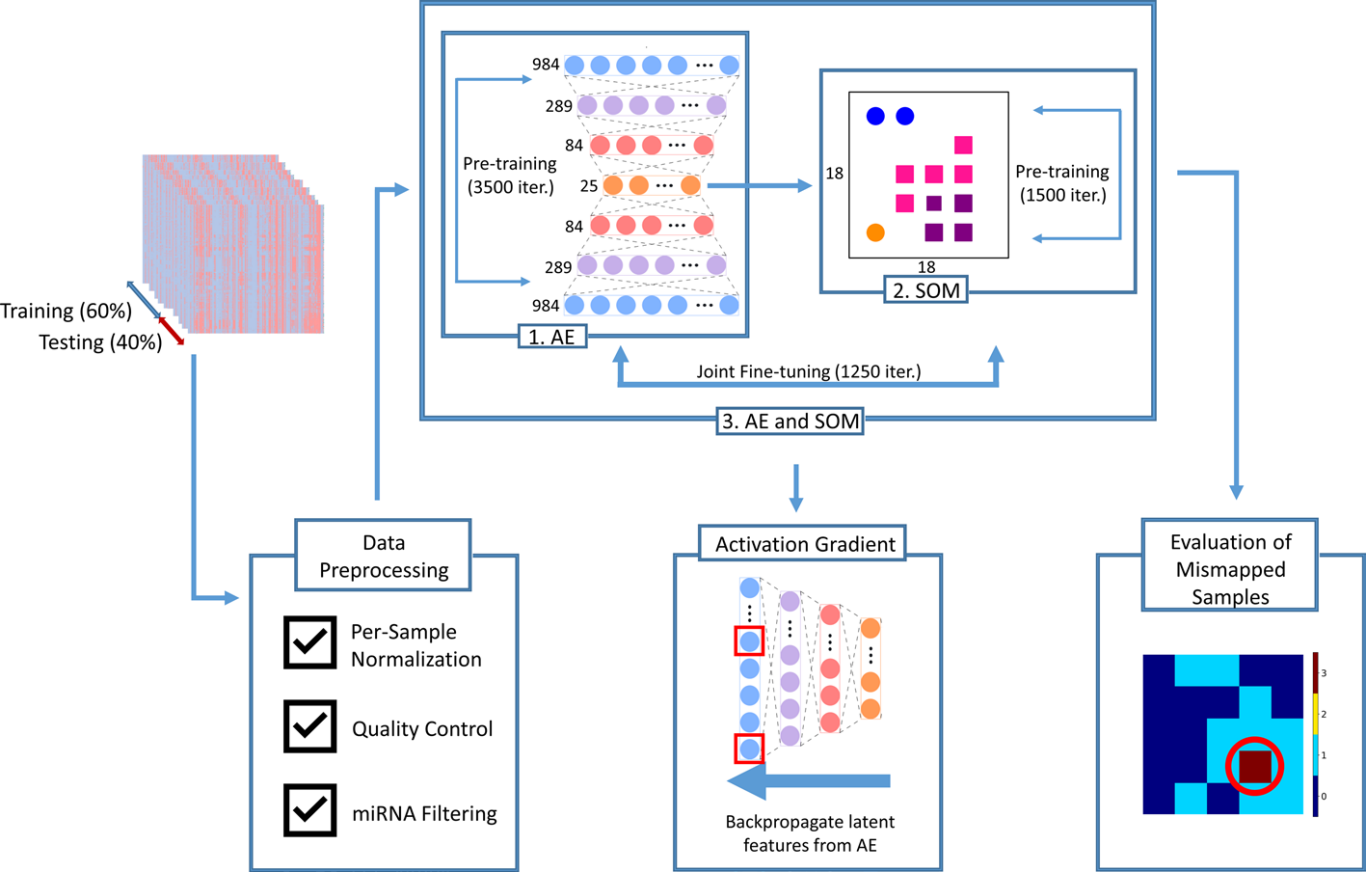
Schematics of the Deep Neural Map, including preprocessing, training, and post-processing. Samples are normalized, outliers are removed, and miRNAs are filtered. Preprocessed training data is the input to a 3-layer symmetric Autoencoder (AE). Once pre-trained, the latent features of the AE are forwarded to the Self-Organizing Map (SOM), which is subsequently pre-trained. Following pre-training of the AE and SOM, joint fine-tuning is performed. Post-processing consists of identification of the attention of the AE to miRNAs through the activation gradient, and identifying samples that do not cluster with their respective class

Our end-to-end unsupervised deep learning approach is shown schematically in Fig. 2 (executed in Google Colaboratory [39]). First, we train an AE to represent the 984 miRNA features of tissue samples as reduced latent features followed by topological mapping of these features to the lattice structure of an SOM. We pre-train the AE to closely recreate the original input using the reduced latent features, indicating accurate representations of the original miRNA profiles. The AE weights are then frozen and the latent features are mapped to a node on the SOM. During pre-training of SOMs, its weights are modified such that samples with similar features are represented by proximal nodes. Next, the AE and SOM weights are jointly fine-tuned to better tailor the AE latent space for specific topological mapping. After training, the ontology of the samples indicating neoplasticity status and tissue-of-origin are used to evaluate if data from the same classes clustered together, and activation gradients of the networks are used to identify most informative miRNAs.

*Autoencoder Pre-Training* To reduce the dimensionality of the input features, the AE is pre-trained as follows. The weights for both the AE and SOM are randomly initialized between 0 and 1. The AE consists of three layers beginning with 984 inputs (corresponding to the filtered miRNAs), and successively reduced number of units in the following layers to form a geometric series leading to the final latent layer. We use Mean Squared Error (MSE) between the input and output of the AE as the loss function, a small regularization term, gradient descent with Adam optimization, a batch size of 64, and 3500 epochs for training.

*Self-Organizing Map Pre-Training* The latent features of the AE are mapped to nodes on a 2D lattice. The weights associated with each node of the lattice are compared to an input sample from the AE using Euclidean distance. The node with the shortest distance to the input sample is labelled as the winning node, and the sample is topologically mapped to that location. During SOM pre-training, the weights of the winning node, and the weights of nodes surrounding the winning node (determined by a topological neighbourhood function) are modified to become more like the input sample. This ensures that similar input samples will eventually be mapped close to each other when training is complete, preserving the topological structure in the data. SOM weights are updated using the difference between the input from AE and the current SOM weights, along with a small learning rate and a Gaussian neighbourhood function. A time-decaying neighbourhood function is used to train the SOM; initially, the neighbourhood is larger to group all similar samples together, and slowly it shrinks to fine-tune the exact location of similar samples to a certain node [34]. A learning rate of 0.005, batch size of 64, and 1500 epochs are used for training the SOM.

*AE and SOM Fine-Tuning* Following pre-training the AE and SOM are jointly fine-tuned. Both the AE and SOM weights are optimized to reduce the distance between the AE output and the winning node of the SOM. For this purpose, the distance of the AE output to the winning node is integrated into the AE loss function as a new term. The SOM weights are updated as explained previously. Joint fine-tuning is run for 1250 epochs. Further details regarding the design, comparison to other methods, and available source code for DNMs can be found at the author’s Github: https://github.com/mpslxz/DNM. In addition, the updated code for implementation using miRNA data, and the addition of activation gradients, can be found at https://github.com/emilykaczmarek/miRNA-DNM.

### Model evaluation

Data are divided into two mutually exclusive sets for training and testing. From the 2524 data samples (1456 neoplastic and 1068 non-neoplastic), 1010 are used for testing and 1514 are used for training. This represents a 40–60% split, respectively, ensuring generalizability of the model while maintaining sufficient training samples to learn data characteristics. In the data division, samples are stratified by combined neoplasticity status and tissue-of-origin class to ensure representation in both training and testing data. A further 20% of the training data are set aside as validation data for hyperparameter tuning. Once the model is trained and parameters chosen using the training and validation data, we use the reserved test data to generate their latent embedding and map tissue samples to the nodes of the SOM lattice. This workflow is unsupervised and the models do not use sample labels at any stage of pre-training or joint optimization.

In order to evaluate model accuracy, the lattice is post-processed where each node is assigned a label of neoplasticity status and tissue-of-origin, determined by the majority of training samples mapped to that location. For nodes that do not have any samples mapped to them, or have a tie between two or more classes, no label is assigned. To determine accuracy, the label of each sample mapped to a certain node is compared to the label of the node. If the labels match, the sample is classified correctly. Otherwise, it is counted as a misclassification. It is important to note the SOM is a topology-preserving cluster method in itself, and the map can be interpreted without labels using previously developed boundary detection algorithms [40, 41]. We choose to interpret the maps by adding labels of the training data post-training, but no labels are ever utilized during model training.

The validation data are used for ablation studies and hyperparameter tuning. The number of training iterations for the AE, SOM, and joint optimization, and the latent size of the AE are chosen based on minimizing the reconstruction error (for the AE) or distance from the winning node (for the SOM) in the validation data.

### miRNA activation gradient

We develop an approach for identifying the attention of the network to inputs and to detect the most informative miRNA in determining a sample’s neoplasticity status and tissue-of-origin. This method is inspired by backpropagation, an approach used for model training in supervised learning to identify weights contributing to incorrect classification. Rather than the gradient of error, the gradient of activations across input nodes of the AE are calculated [33, 42]. For every weight connecting two nodes in the AE, a signed activation gradient can be calculated. The contribution of a specific input node to a latent node in the AE can be determined by the sum of the absolute values of the activation gradients for all weights connected to the input node. A distinct activation is produced in response to groups of samples (e.g. those from one tissue-of-origin and neoplasticity status) yielding “input activation gradients” per class. The miRNAs that contribute the most to the latent features of the AE for samples of a given tissue and neoplasticity status (i.e., those with highest activation gradient) are the most informative miRNAs to this class.

### Analysis of multi-class SOM nodes

For nodes on the SOM lattice that have samples from two or more classes mapped to them, further analysis is performed to identify the source of the apparent improper groupings. First, locations with multiple classes are identified. The activation gradient is used to determine the significant miRNAs in the specific samples mapped to these locations, as opposed to the average per-class activation described in the previous section. We refer to the terminology as “sample-specific activation” and “class-average activation”, respectively. For example, if neoplastic breast, skin, and thyroid samples are mapped to one node, the specific samples are used to calculate sample-specific activations. Any miRNAs identified as highly activated in all three of the classes are noted and pursued through literature for known biochemical roles. We only study a subset of highly activated miRNAs and limit them to those contributing to a total of 75% of activation gradients in their corresponding class. In addition, any miRNAs that contribute highly to the class-average activations of these samples but do not appear in the sample-specific activations are also studied.

## Results

As mentioned in Methods, the Deep Neural Map is analyzed to determine its ability to topologically map similar samples to spatially proximal nodes, solely based on their molecular profile. Specifically, after unsupervised learning, labels are used to post-process the SOM lattice with respect to the mapping of neoplastic and non-neoplastic samples, as well as the tissue-of-origin. We use accuracy as the quantitative metric to assess performance, calculated for neoplasticity status, tissue-of-origin, and combined neoplasticity status and tissue-of-origin on held-out test data, as well as sensitivity and specificity for the neoplasticity status of samples. We implement the miRNA activation gradient approach to identify miRNAs that have significant roles in mapping and stratifying the samples. We then study the clusters of samples in topological maps and calculate an average activation for miRNAs per class. Comparison of the average activations between neoplastic and non-neoplastic samples from the same tissue-of-origin allows identification of potential cancer biomarkers. Lastly, nodes on the SOM that have multiple classes mapped to them are analyzed with the miRNA activation gradient to determine sources of errors in clustering.

### Classification accuracy

**Fig. 3:**
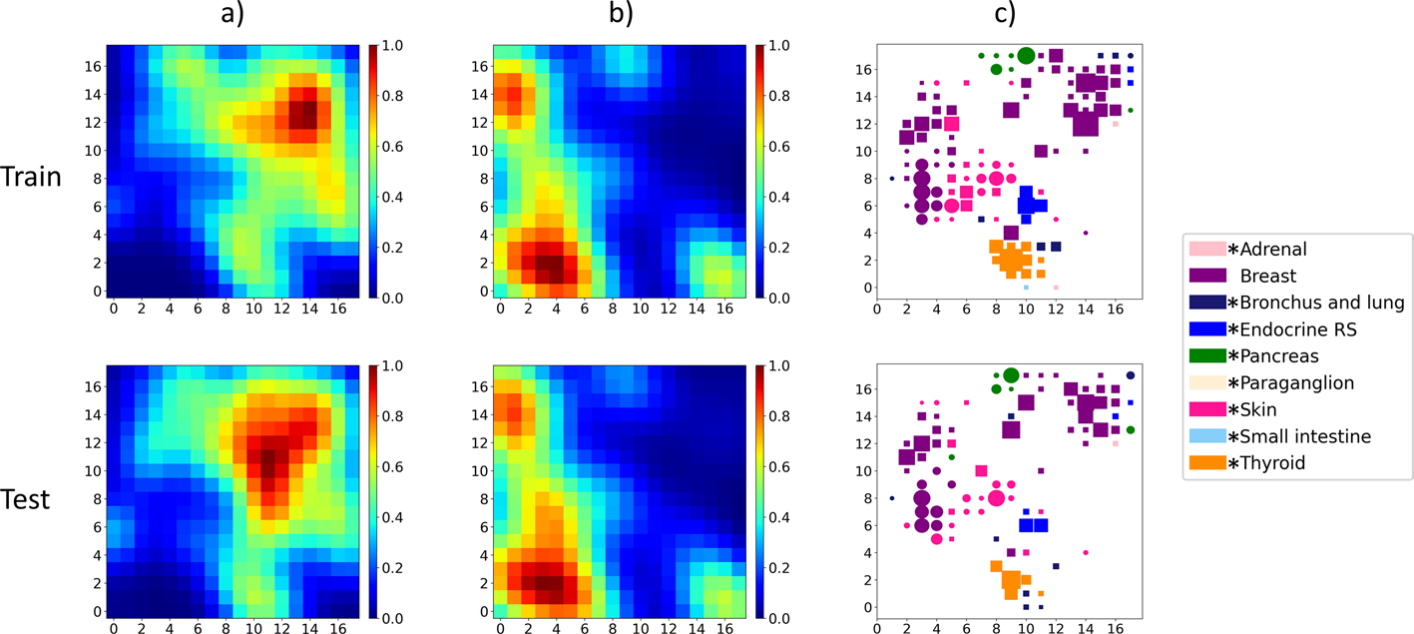
SOM lattice showing mapping of all **a** neoplastic tissue, **b** non-neoplastic tissue, and **c** neoplasticity status and tissue-of-origin from nine selected tissues-of-origin for both training (top) and test (bottom) data. The x and y axes represent SOM coordinates. The color of the heatmaps indicates the density of samples in columns **a**, **b**. In column **c**, circles and squares represent non-neoplastic and neoplastic samples, respectively, with the size of the marker proportional to the number of samples of the classes mapped to that location. From columns **a**, **b**, the DNM is able to stratify neoplastic and non-neoplastic tissue through mapping to different areas of the SOM. Clear cluster-centers can be seen in both of these images in red. Column **c** shows clear clusters of similar tissue and neoplasticity status. Models generalize well to test data as seen from the top and bottom rows of the figure. In the legend, * indicates that these neoplastic tissue types consist of some/all neuroendocrine tumour samples

The topology maps representing the stratification of the data using the learned models are shown in Fig. 3 as heatmaps. Column (a) represents the heatmap of all neoplastic samples (training and test data separately), with the colour representing the density of samples mapped to each location. Column (b) shows the heatmap of all non-neoplastic samples mapped to the lattice. As seen, the neoplastic and non-neoplastic samples form clear clusters that are far apart in the lattice with centres of the clusters shown in red. Column (c) shows a subset of samples mapped to the SOM lattice but annotated with tissue-of-origin as well as neoplasticity status information. For simplicity we show the lattice with data from nine of the total tissue classes. In this column, circles represent non-neoplastic samples while rectangles are neoplastic, color corresponds to the tissue-of-origin and the size of the marker is proportional to the number of samples of the classes mapped to that location. For instance, purple circles and squares show normal and cancer breast samples. For tissues where corresponding cancer or normal samples are not visible, they were mapped to locations with predominantly another tissue type and not assigned a node label. The results in the top and bottom row of Fig. 3 indicate that the models generalize well outside of the training data with the test data maps following similar patterns.

The DNM is able to cluster samples from the same class, i.e., same tissue type and neoplasticity status, with 84.24% accuracy as shown in Table 1. When examining only the classification of the neoplasticity status of samples, i.e., cancer and benign tissue, the accuracy increases to 91.07%, with sensitivity and specificity of 94.70% ($+/-$ 0.774) and 92.97% ($+/-$ 0.801), respectively. However, the DNM can only cluster the tissue-of-origin of samples with an accuracy of 86.36%. This is subsequently compared to a Multi-layer Perceptron (MLP), with corresponding accuracies shown in Table 1. The MLP has highest accuracy for each of neoplasticty status, tissue-of-origin, and combined neoplasticity status and tissue-of-origin. The DNM and MLP are each run 10 times, with the mean accuracy and standard deviation displayed in Table 1. Since the visualization of the SOM changes with each run due to random initialization, we present all results other than the classification accuracies (confusion matrix, visualization, activation gradients, and interpretation of misclassifications) corresponding to one of the ten trials. However, the results between trials did not have many differences (e.g., the miRNAs identified by the activation gradient largely remained constant throughout the repeated trials).

**Table 1:** Classification accuracy of Deep Neural Map and Multi-Layer Perceptron for both combined and individual neoplasticity status and tissue-of-origin of samples

Trial	Combined neoplasticity status and tissue-of-origin	Tissue-of-origin	Neoplasticity status
DNM	84.28% (1.48)	86.36% (1.58)	91.07% (0.93)
MLP	93.28% (0.16)	95.23% (0.14)	96.26% (0.14)

The standard deviation of 10 trials is shown in brackets

**Fig. 4:**
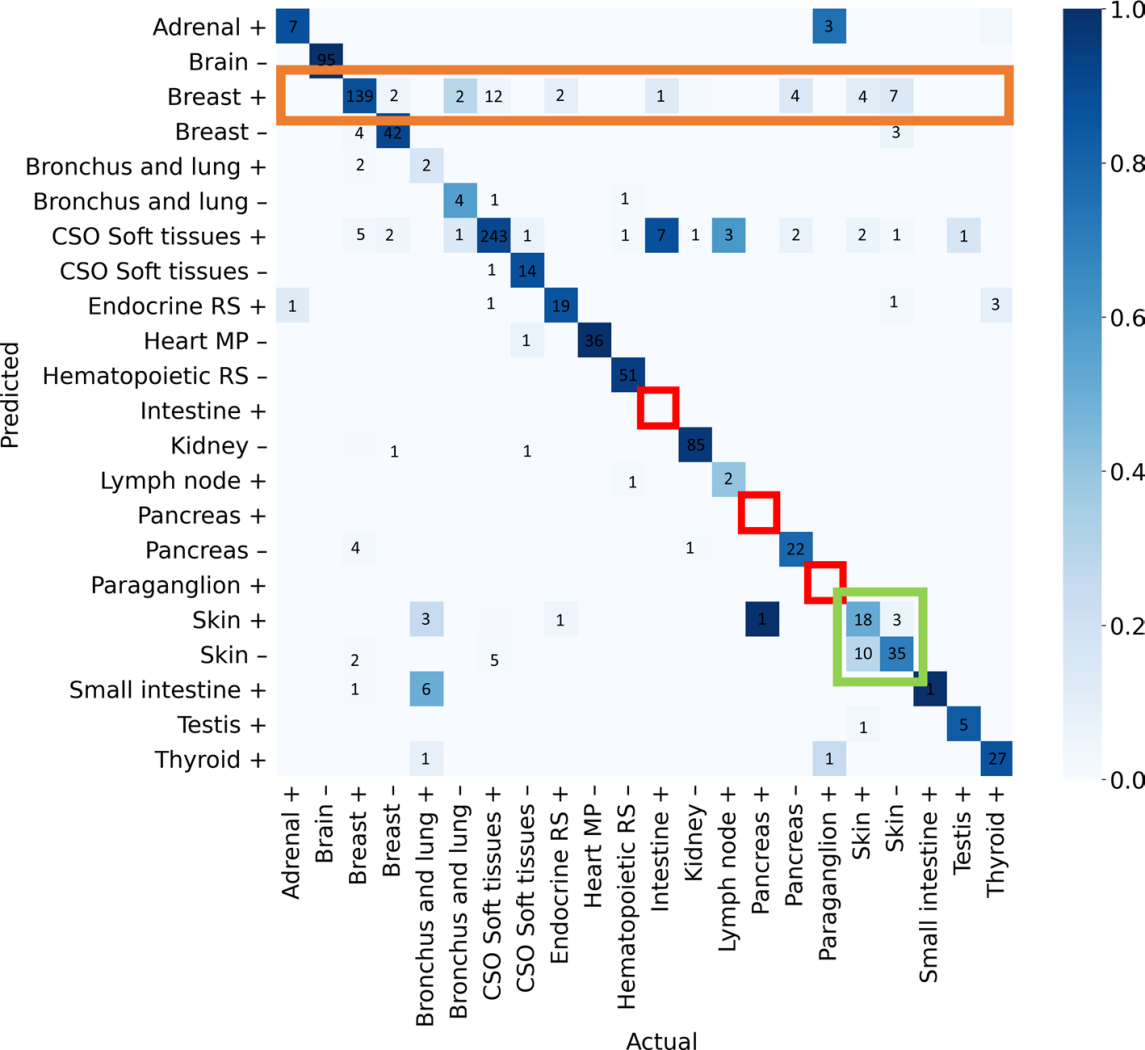
The confusion matrix of classification of samples for combined tissue-of-origin and neoplasticity status, in test data. The colour of each square is proportional to the number of samples classified as the label of that square. The diagonal of the matrix is dark, indicating a high number of correctly classified samples. Non-neoplastic classes generally have fewer misclassifications. A number of neoplastic skin tissue samples are misclassified as non-neoplastic skin tissue (green). Multiple classes are predicted to be neoplastic breast tissue (orange). Three classes are identified in red as not having any nodes assigned them. Above, + represents neoplastic tissue, and − represents non-neoplastic tissue

The confusion matrix depicting the classification results for joint neoplasticity status and tissue-of-origin is in Fig. 4 as a heat map. As seen, the diagonal of the matrix is dark indicating a large proportion of correctly classified samples. There are a high number of misclassifications between non-neoplastic and neoplastic skin tissue (green square), and between neoplastic breast tissue and other classes (orange rectangle). In addition, classes that do not have a majority of samples at any SOM node (resulting in no ‘actual’ nodes of that label) are highlighted (red squares). From the confusion matrix, non-neoplastic tissue is generally misclassified less frequently than neoplastic tissue.

### miRNA activation gradient

**Fig. 5:**
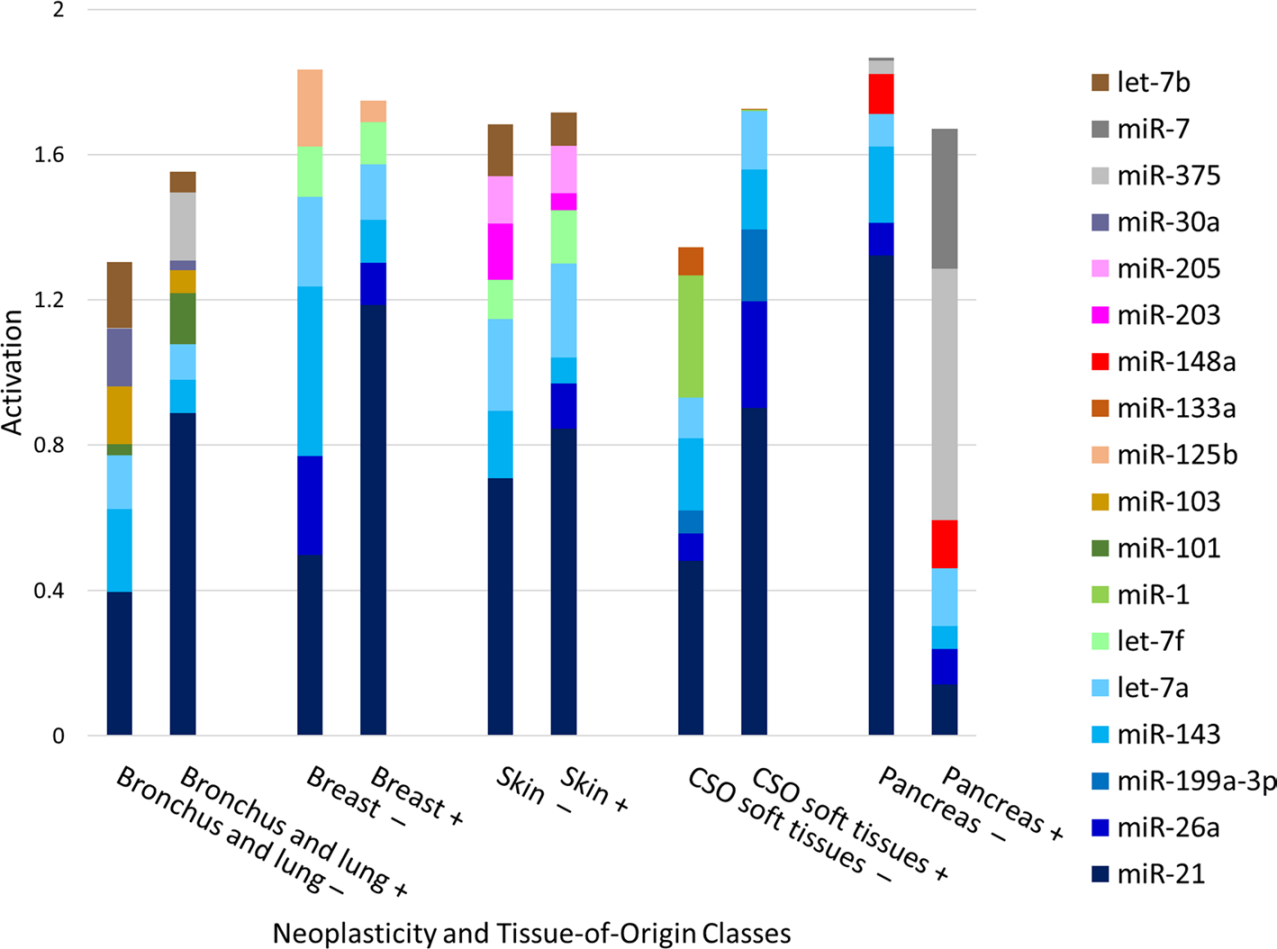
Comparison of the most activated miRNAs, identified by the activation gradient for neoplastic and non-neoplastic tissues of the same origin. Tissue classes all share 4–5 highly activated miRNAs, seen in the bottom of each column. Near the top of each column, unique miRNAs are seen which can distinguish neoplasticity status and/or tissue-of-origin. In the labels, + represents neoplastic tissue, and − represents non-neoplastic tissue

A summary of the results from the implemented miRNA activation gradient is shown in Fig. 5. Ten combined neoplasticity status and tissue-of-origin classes are displayed, chosen specifically to show the difference between neoplastic and non-neoplastic activation of tissue from the same origin. To simplify the analysis, the top five miRNAs identified by the activation gradient for each class are chosen. The activations of those miRNAs are depicted for both neoplastic and non-neoplastic samples of a tissue class. In other words, for each pair of neoplastic and non-neoplastic tissue, activations from a minimum of five miRNAs are shown. This enables direct comparison of miRNA activations, where differences between neoplastic and non-neoplastic tissue can lead to identification of potential biomarkers. All classes share 4–5 similar highly activated miRNAs, shown at the bottom of the columns in Fig. 5. Moving upwards, several unique miRNAs are seen, which are specific to neoplasticity status and/or tissue-of-origin.

### Analysis of multi-class SOM nodes

**Fig. 6:**
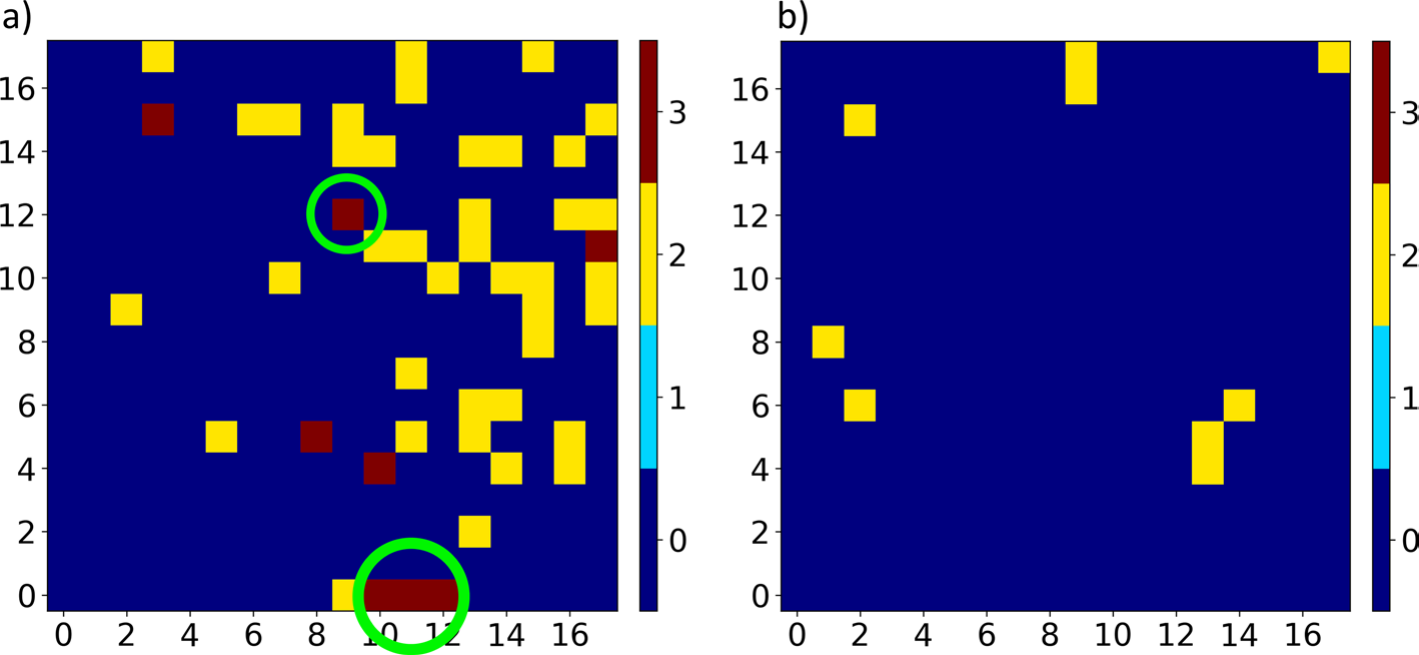
Heatmaps showing nodes with two or more neoplastic classes (**a**) or non-neoplastic classes (**b**) mapped to them. The x and y axes represent SOM coordinates. Non-neoplastic samples have fewer misclassifications. Nodes with two classes are likely transition nodes between two clusters on the SOM. Multi-class node analysis is thus limited to nodes with three or more classes. Four of these nodes (green, **a**) are examined in Table 2

Samples that are mapped to nodes with two or more class labels are examined. The miRNA activation gradient is used to determine the similarities between informative miRNAs in these samples. Figure 6 identifies the nodes in the SOM lattice with more than one class mapped to them. Nodes with multiple neoplastic-class samples mapped to them are shown in Fig. 6a, and those with multiple non-neoplastic-class samples are shown in Fig. 6b. Non-neoplastic samples cluster together with fewer misclassifications than neoplastic samples. Only nine nodes in the lattice have two classes of non-neoplastic tissue. Locations on the lattice that have representatives from two classes are likely transition nodes between two distinct spatial clusters. Multi-class node analysis is thus confined to nodes with three or more classes. Four nodes (highlighted in green in Fig. 6a) with at least three classes are examined further with the miRNA activation gradients. These results are shown in Table 2. The location of the node (matched to Fig. 6a) and classes mapped to them are in the first two columns, with the number of samples in each class shown in brackets. Key miRNAs shared between all sample-specific activations are shown in column 3, while those different between class-average activations and sample-specific activations are shown in column 4. Arrows are used in columns 3 and 4 with a miRNA if all sample-specific activations of that miRNA are above or below their respective class-average. For example, in the first row, all sample-specific activations of miR-375 are higher than the class-average activation of this miRNA for each of the neoplastic small intestine, pancreas, and bronchus and lung classes. Therefore, miR-375 has its activation trend in this node shown with an up arrow. However, for miR-7, the sample-specific activations in those three classes are mixed between being either higher or lower than their respective class-average activations. Therefore miR-7 does not have a consistent trend in all samples mapped to that node and is shown without an arrow. miRNAs without an arrow are still important shared properties of the samples mapped to the same location, but common activation trends are not shared between them.

**Table 2:** Analysis of nodes with more than two classes, corresponding to the two areas circled in green in Fig. 6

Location	Classes	Shared miRNAs	Shared miRNAs in class-average activations
[10,0]	Neoplastic small intestine (3), Neoplastic pancreas (1), Neoplastic bronchus and lung (2)	miR-375 $\uparrow$, miR-7, miR-26a, let-7f, let-7a $\downarrow$	
[11,0]	Neoplastic small intestine (4), Neoplastic pancreas (4), Neoplastic bronchus and lung (3)	miR-375, miR-7, miR-26a, let-7a, let-7f $\uparrow$	
[12,0]	Neoplastic adrenal gland (6), Neoplastic pancreas (1), Neoplastic paraganglion (5)	miR-7 $\uparrow$, miR-375, let-7a, miR-26a, let-7f	
[9,12]	Neoplastic connective, subcutaneous, and other soft tissues (9), Neoplastic skin (1), Neoplastic breast (1)	miR-21 $\uparrow$, miR-26a $\downarrow$, miR-199a-3p $\uparrow$	miR-125b $\downarrow$

The first two columns show the lattice nodes and classes mapped there, with the number of samples in each class shown in brackets. The third column shows highly-activated key miRNAs shared between the sample-specific activations (within the top 75% of contributing miRNAs per class). The final column displays key miRNAs common between class-average activations, but not found within any of the sample-specific activations. Up (or down) arrows represent any miRNAs where all sample-specific activations of that miRNA are higher (or lower) than their respective class-average value

## Discussion

While comprehensive molecular characterization will transform our understanding of cancer, advanced computational methods are also needed to generate insights from high-dimensional datasets. To address this need, we develop a novel approach using DNM for end-to-end unsupervised representation and visualization of neoplastic and non-neoplastic tissue samples that allows their molecular properties to directly determine their similarity. Briefly, our approach involves mapping a topological distribution of neoplastic and non-neoplastic samples to an SOM, identifying samples that do not cluster with their class, and using activation gradients to better understand the mechanistic significance of select miRNAs. Combining the identification of misclassified samples on the SOM lattice and the interpretation of the significance of miRNA profiles in their representation, leads to better understanding of cancer. In particular, samples that do not cluster well with others with similar ontology are analyzed for differences in their miRNA expression. This has the potential to reveal biomarkers shared between cancers of different origin, and possible targeted treatment options that are patient-specific.

**Fig. 7:**
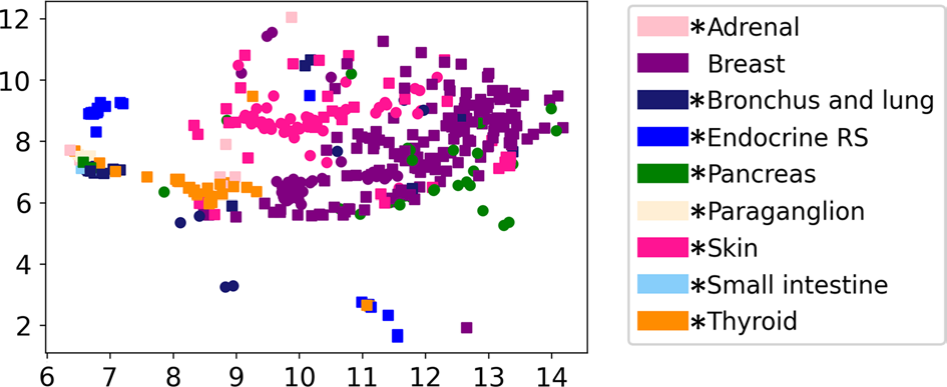
UMAP visualization of test data, displaying the same classes as the DNM in Fig. 3. In this figure, circles and squares represent non-neoplastic and neoplastic tissue, respectively. In the legend, * indicates that these neoplastic tissue types consist of some/all neuroendocrine tumour samples

*Comparative Approaches and Ablation Study* DNM provides an unsupervised approach for visualizing data clusters based on their inherent properties. Comparisons to other well-known visualization techniques have been reported previously in Pesteie et al. [34]. As opposed to Principal Component Analysis (PCA) [43] which reduces dimensionality linearly, the DNM is non-linear. Compared to t-distributed Stochastic Neighbor Embedding (t-SNE) [44] and Uniform Manifold Approximation and Projection (UMAP, Fig. 7) [45], our solution fine-tunes the latent space in DNMs to provide better topological mapping and low reconstruction error by integrating the SOM and AE losses. In addition, our solution can embed data into a larger latent space than t-SNE and UMAP, preserving the topological structures among data points. This allows the optimization of the latent space, preventing the loss of important features. We experiment with various dimensions of the AE and hyperparameters for achieving the best reconstruction error, and try the range of 10–40 for the latent space. The AE loss decreases while the latent space grows to 25 and increases afterwards. We also notice that as the loss increases, samples of the same class are more dispersed on the lattice, showing the importance of optimizing the latent size. This highlights one of the advantages over traditional visualization techniques; the SOM is able to map higher dimensional features in two dimensions while preserving the topology. The lower error using a latent space of 25 indicates that UMAP and t-SNE may lose important information during feature reduction, leading to less refined clusters.

*DNM Clustering and Accuracy* The DNM is able to effectively discriminate between neoplastic and non-neoplastic tissues. Distinct clusters of tissue samples are formed, corresponding to these designations, on opposite sides of the lattice in Fig. 3a, b. This suggests that cancers of different tissue-origins share sufficient miRNA features that are specific to neoplasticity. This observation has been previously reported in the literature, e.g., miR-21 is shown to be up-regulated in many cancers [46]. This is further seen by the high accuracy of classifying neoplastic and non-neoplastic samples (Table 1). The DNM also results in distinct clusters of samples when examining combined neoplasticity and tissue-of-origin, shown in Fig. 3c. The presence of distinct clusters of tissues-of-origin demonstrates the known ability of miRNAs to be used for tissue typing [9]. The DNM is able to map the held-out test data to similar locations as their respective classes seen during training and generalizes well to unseen data. The DNM accuracy is next compared to a supervised MLP. The MLP outperforms the DNM in all three classification tasks. The MLP uses the labels of each sample during training, whereas the DNM uses only similarity measures to identify classes. While two samples may have the same neoplasticity status and tissue-of-origin, the true miRNA dysregulation and/or molecular properties may differ largely, leading to misclassifications. The motivation behind the DNM is to identify the most similar samples based purely on molecular properties, and analyze samples that do not cluster well with their respective class, leading to better understanding of individual patient samples. This is performed by analyzing samples mapped to specific nodes, as opposed to analyzing clusters of multiple nodes. Samples at the same nodes are the most similar to each other; it is therefore expected that these would be samples from the same class. Those that differ from the expectation are potentially abnormal samples which should be further analyzed. While these are called ‘misclassifications’, they may represent samples with unusual miRNA dysregulation.

*DNM Misclassifications* Our method allows the further exploration of misclassified samples quantitatively. A large number of neoplastic skin samples are misclassified as non-neoplastic skin (Fig. 4, green), which is also shown through their proximal mapping in Fig. 3c. Looking at the class-average activation gradients, these two classes have many similarly activated miRNAs (Fig. 5), resulting in challenges determining class differences. An additional issue contributing to such challenges is that skin samples obtained for pathology contain multiple different cell types, leading to varying molecular signatures.

Multiple classes of tissue were wrongly mapped to nodes labeled as neoplastic breast (Fig. 4, orange). Neoplastic breast samples share some of their most activated miRNAs (e.g. miR-21, miR-26a) with numerous classes, seen in Fig. 5. In addition, miRNA expression is more varied within neoplastic tissue due to differing cancer grade and subtypes [31, 47], which may contribute to more misclassifications. This is further seen through the high number of multi-class neoplastic nodes shown in Fig. 6a compared to the number of non-neoplastic multi-class nodes in Fig. 6b. The low number of non-neoplastic multi-class nodes displays the stable expression of healthy tissue and acts as a positive control for the DNMs (Fig. 6).

A challenge we face is that for classes with low number of samples and heterogenous cell types, e.g. neoplastic intestine tissue (intestine samples share smooth muscle features with other soft tissues), the DNM is not able to map them to designated nodes. Instead they are often misclassified as other classes they may share features with (Fig. 4, red). Through identifying these misclassifications, it is possible to further study molecular similarities between known classes or discover new shared signatures.

**Fig. 8:**
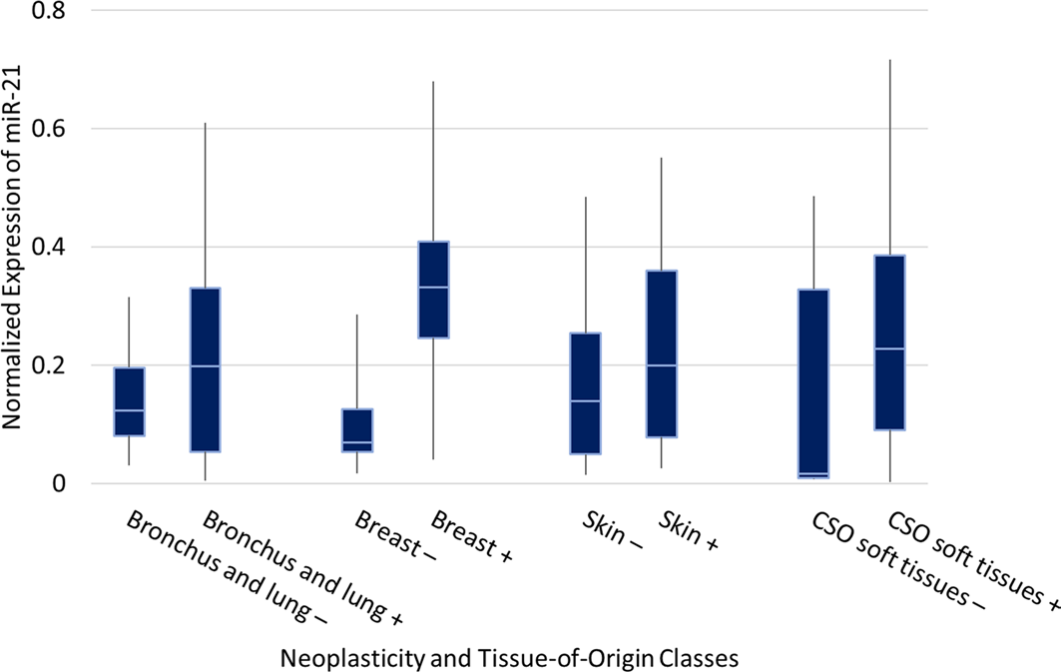
Boxplot displaying the RF normalized expression of miR-21 for four tissues-of-origin with both neoplastic and non-neoplastic tissue samples. The initial expression of this miRNA is examined after the activation gradients identified it as significant for classification of tissue-of-origin and neoplasticity

*Discovery of Cancer Biomarkers* The miRNA activation gradients provide a novel approach for proposing potential cancer biomarkers and molecular drivers through comparison of neoplastic and non-neoplastic activation for the same organ. From Fig. 5, in all classes except pancreatic tissue, miR-21 has higher activation in neoplastic than non-neoplastic tissue. This difference suggests miR-21 could be an important biomarker for discriminating neoplastic and non-neoplastic tissue in these organs. We then examine the initial normalized expression of miR-21 in these tissues in Fig. 8, which indeed shows a difference in expression between neoplastic and non-neoplastic tissue in each organ. In addition, miR-21 is a known oncomiR for certain cancers shown to have upregulated expression, which has been linked to overtargeting of genes that prevent metastasis and apoptosis [46, 48]. Many other potential biomarkers in Fig. 5 have also been reported in the literature. For example, miR-143 has lower activation in neoplastic bronchus and lung, breast, and skin tissue compared to its respective non-neoplastic activations. miR-143 is a known tumour-suppressor, and its down-regulation has already been linked to many cancers [49]. Further, let-7b has lower activation in neoplastic bronchus and lung, which is a known tumour-suppressor and has been shown to target KRAS. The KRAS gene is often mutated in lung cancer, which can prevent binding of let-7b to the target site (preventing mRNA degradation), and downregulate let-7b through further downstream complications of upregulated KRAS [50]. This is a key example of how computation meets biology and identifies important parts of entire regulatory networks. While our proposed approach can be used to identify potential cancer biomarkers, a knowledge of miRNA tissue specificity is essential to prevent the misidentification of tissue-specific markers as cancer biomarkers [9]. For example, miR-1 appears to have low activation in neoplastic soft tissue samples. This is likely due to how the tissue samples were collected. miR-1 is a known muscle tissue marker; during sample collection, the non-neoplastic samples likely contained muscle tissue, whereas the neoplastic samples collected tissue only from the tumour. The heterogeneity of sample collection must be considered when examining potential miRNA biomarkers. In addition, while activation partly reflects the expression of a miRNA, its purpose is to highlight the attention of the deep learning model to this particular marker.

*Multi-Class Node Interpretation—Case 1* To further examine misclassified samples we study multi-class nodes of the SOM lattice that have three or more classes mapped to them. Upon analysis of nodes [10,0], [11,0], and [12,0] (Fig. 6a, green, and Table 2), it is found that the apparent misclassifications at the nodes are the result of neuroendocrine tumour samples. Neuroendocrine tumours (NETs) are a rare form of cancer that develop within the neuroendocrine cells of numerous different organs [51]. In the dataset we use, NETs are present in a total of 8 of the 17 tissue types (adrenal gland, bronchus and lung, endocrine and related structures, pancreas, paraganglion, skin, small intestine, and thyroid gland). However, the NETs are only annotated in the disease subtype, which we do not consider in this study. Calculating sample-specific activation gradients at these three nodes indicates shared features of miR-375 and miR-7, which are specific to NETs [21]. Upon closer evaluation of the disease subtypes of the samples, every sample mapped to these nodes is indeed a NET. Therefore, although these samples are from different organs and have distinct molecular profiles, the DNM is able to identify signatures specific to NETs, and map these samples to the same location.

Since miRNAs specific to NETs are significantly represented in our data, we analyze the remainder of NETs and found that while these samples cluster with other samples from their respective tissue-of-origin, the majority also cluster together at the bottom of the SOM (Fig. 3c). Using class-average activation gradients we also identify that both neoplastic pancreas and neoplastic bronchus and lung have high activation of miR-375, with neoplastic pancreas having miR-7 as well (Fig. 5).

*Multi-Class Node Interpretation—Case 2* Node [9,12] shown in Fig. 6 and Table 2, represents samples from soft tissue, breast and skin cancers. We calculate sample-specific activation gradients for these cases and compare them with their corresponding class-average activations. We found that several shared miRNAs in these samples have significantly higher or lower activations when compared to class-averages. Selected shared features include high activation of miR-21 and miR-199a-3p, and low activation of miR-26a and miR-125b. These miRNAs are known oncomiRs (miR-21, miR-199a-3p) and tumour suppressors (miR-26a, miR-125b), and also identified by our models as significant contributors to abnormal sample clustering [12, 52, 53]. It is possible that all these samples are those of higher grade or aggressiveness compared to others from their respective class in the data. We hypothesize that it is possible to use the SOM maps to identify potential higher grade tumours or other abnormalities in patient-specific samples, a likely valuable tool for experimental cancer biologists.

## Conclusion

Cancer classification is an important step to determine proper treatment and improve prognosis. The use of miRNAs as cancer biomarkers is becoming increasingly common; however, the understanding of the role of miRNAs in cancer can still be improved. In this study, we present an end-to-end unsupervised solution that can stratify tissue types using miRNA expression data. A Deep Neural Map is created, showing clusters of tissue-of-origin and neoplasticity status of samples. In addition, a method based on the activation gradient of the AE network is implemented to determine the miRNAs contributing most to the stratification of each class. Importantly, this method is applied to study misclassifications on the Deep Neural Map. In understanding the irregular miRNA expression of misclassified samples, better patient-specific classification and treatment can be determined. Future work will include experimenting with other architectures of the Deep Neural Map that incorporate different autoencoders and distance metrics. A cost-sensitive penalty factor could be incorporated in attempt to minimize misclassifications. In addition, the top miRNAs identified from the activation gradients should be used as input to a DNM model as opposed to all miRNAs, to determine the effect on clustering. We also hope to extend this work to include other types of data such as mRNA, genome sequencing, and mass spectrometry for proteins, particularly in cases where potential biomarkers are underexplored. This can lead to further insight on associations and similarities between tissue samples as well as the process of gene dysregulation, and how each of these molecules interact with each other (i.e., pathway analysis).

## Data Availability

A portion of the breast cancer dataset and neuroendocrine tumour/matched healthy samples analysed during the current study are available in the Supplementary material of Farazi et al. [36] and in Data Dryad (https://datadryad.org/stash/dataset/doi:10.5061/dryad.fn2z34tqj). The remaining datasets analysed during the current study are not publicly available due to being part of an ongoing comprehensive microRNA sequence reannotation project and will be released in the near future.
